# Analysis of MIR27A (rs11671784) Variant Association with Systemic Lupus Erythematous

**DOI:** 10.3390/life13030701

**Published:** 2023-03-05

**Authors:** Zenat Ahmed Khired, Shahad W. Kattan, Ahmad Khuzaim Alzahrani, Ahmad J. Milebary, Mohammad H. Hussein, Safaa Y. Qusti, Eida M. Alshammari, Eman A. Toraih, Manal S. Fawzy

**Affiliations:** 1 Department of Surgery, College of Medicine, Jazan University, Jazan 45142, Saudi Arabia; 2 Department of Medical Laboratory, College of Applied Medical Sciences, Taibah University, Yanbu 46423, Saudi Arabia; 3 Medical Laboratory Technology, Faculty of Applied Medical Sciences, Northern Border University, Arar 91431, Saudi Arabia; 4 Department of Medical Laboratory, King Fahad Armed Forces Hospital, Jeddah 23311, Saudi Arabia; 5 Division of Endocrine and Oncologic Surgery, Department of Surgery, Tulane University School of Medicine, New Orleans, LA 70112, USA; 6 Department of Biochemistry, Faculty of Science, King Abdulaziz University, Jeddah 21589, Saudi Arabia; 7 Department of Chemistry, College of Sciences, University of Ha’il, Ha’il 2440, Saudi Arabia; 8 Medical Genetics Unit, Department of Histology and Cell Biology, Suez Canal University, Ismailia 41522, Egypt; 9 Department of Medical Biochemistry and Molecular Biology, Faculty of Medicine, Suez Canal University, Ismailia 41522, Egypt; 10 Department of Biochemistry, Faculty of Medicine, Northern Border University, Arar 1321, Saudi Arabia

**Keywords:** MIR27A, SLE, gene variant, polymorphism, Real-Time PCR, rs11671784

## Abstract

Multiple microRNAs (miRs) are associated with systemic autoimmune disease susceptibility/phenotype, including systemic lupus erythematosus (SLE). With this work, we aimed to unravel the association of the miR-27a gene (MIR27A) rs11671784G/A variant with SLE risk/severity. One-hundred sixty-three adult patients with SLE and matched controls were included. A TaqMan allelic discrimination assay was applied for MIR27A genotyping. Logistic regression models were run to test the association with SLE susceptibility/risk. Genotyping of 326 participants revealed that the heterozygote form was the most common genotype among the study cohort, accounting for 72% of the population (n = 234), while A/A and G/G represented 15% (n = 49) and 13% (n = 43), respectively. Similarly, the most prevalent genotype among cases was the A/G genotype, which was present in approximately 93.3% of cases (n = 152). In contrast, only eight and three patients had A/A and G/G genotypes, respectively. The MIR27A rs11671784 variant conferred protection against the development of SLE in several genetic models, including heterozygous (G/A vs. A/A; OR = 0.10, 95% CI = 0.05–0.23), dominant (G/A + G/G vs. AA; OR = 0.15, 95% CI = 0.07–0.34), and overdominant (G/A vs. A/A + G/G; OR = 0.07, 95% CI = 0.04–0.14) models. However, the G/G genotype was associated with increased SLE risk in the recessive model (G/G vs. A/A+ G/G; OR = 17.34, 95% CI = 5.24–57.38). Furthermore, the variant showed significant associations with musculoskeletal and mucocutaneous manifestations in the patient cohort (*p* = 0.035 and 0.009, respectively) and platelet and white blood cell counts (*p* = 0.034 and 0.049, respectively). In conclusion, the MIR27A rs11671784 variant showed a potentially significant association with SLE susceptibility/risk in the studied population. Larger-scale studies on multiethnic populations are recommended to verify the results.

## 1. Introduction

Systemic lupus erythematosus (SLE; OMIM 152700) is a prototypic autoimmune complex disease that is characterized by excessive production of autoantibodies against a broad range of self-antigens [1,2]. Vital organs and tissues are often affected, including the kidney, brain, cardiovascular system, joints, and skin [3]. The pathogenesis of SLE is complex, with evidence of genetic/epigenetic–environment interplay shaping the clinical variability of such a disorder [4,5]. Unraveling the disease susceptibility and phenotype-associated genetic markers is a crucial step toward precision medicine in SLE [6].

MicroRNAs (miRNAs) are a family of noncoding RNAs (ncRNAs) transcribed by RNA polymerase II into primary transcripts (pri-miRNAs) that are then cleaved to form hairpin precursor miRNAs (pre-miRNAs) of 70–100 nucleotides, with the aid of Drosha [7]. These hairpins subsequently undergo further processing by the endonuclease enzyme Dicer, yielding a duplex of 19–22 nt. One strand of the duplex is integrated into the RNA-induced silencing complex and delivers mature miRNAs to the respective mRNA targets [8]. By mediating mRNA degradation and/or translation inhibition through canonical and non-canonical mechanisms, miRNAs play central roles in gene regulation [7]. One of the essential characteristics of miRNAs is their export and migration from their host cells, where they are transcribed/processed into several body fluids, including the blood (circulating miRNAs) in highly stable forms due to their inclusion in the exosomes and/or interaction with several circulating proteins, such as argonaute 2 and nucleophosmin 1, and high-density lipoproteins that protect them from degradations by RNases [9]. This class of ncRNAs, as regulators of post-transcriptional gene expression, has been implicated in several physiological process and pathological disorders, including SLE [10,11,12,13,14,15,16,17,18,19,20,21,22].

**Figure 1:**
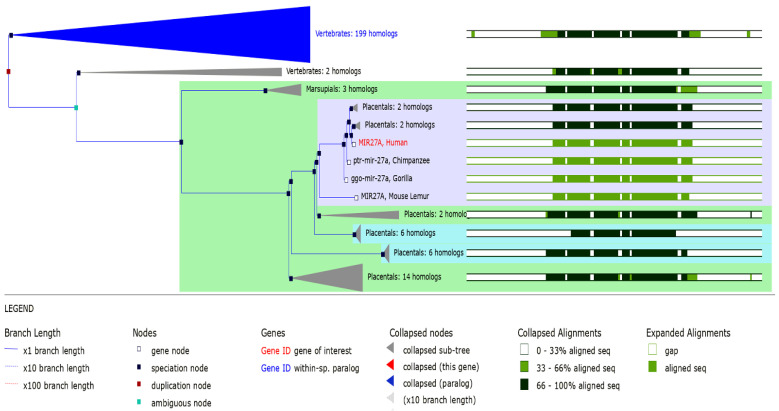
GeneTree (RF00644) of human MIR27A showing conservation across vertebrates. All of the sequences are only involved in differences in 3′ ends, with fewer detected varied nucleotides. Homologous miRNAs also detect common core sequences (data source: http://asia.ensembl.org/ (accessed 20 December 2022)).

MicroRNA-27a (miR-27a) has been identified to be highly conserved throughout vertebrate genomes during evolution (Figure 1) and is considered a member of the miRNA-23∼27∼24 cluster, with several essential biological roles [23,24].

Accumulating evidence indicates miR-27a implication in the pathogenesis of SLE, which can be identified as a potential biomarker for SLE due to its ability to regulate the expression of genes associated with disease phenotypes [25,26,27,28]. Guttilla and colleagues reported that miR-27a, along with miR-96/miR-182, downregulated the transcriptional factor “FOXO-1”, which regulates genes implicated in apoptotic response, cell metabolism, and cell cycle checkpoints [29]. Interestingly, FOXO-1 transcript levels were downregulated in the peripheral blood mononuclear cells (PBMCs) of SLE patients with active disease and were inversely correlated with lupus disease activity [30]. Furthermore, Tardif et al. observed that miR-27a could indirectly downregulate the “matrix metalloprotease-13 (MMP-13) and the insulin-like growth factor binding protein (IGFBP)”, two genes implicated in osteoarthritis [31]. In an independent study, Lin and colleagues reported that miR-27a could block PPARγ transcriptional induction [32]. This factor has been implicated in the etiopathology of several diseases [25], including SLE, as upregulated PPAR-γ was found to modulate monocytes into an M2-like phenotype in patients with SLE [33]. Collectively, it appears that miR-27a could play essential roles in SLE pathology and phenotype and be a novel therapeutic target.

Regarding dysregulated levels of miR-27a in SLE, Sourour et al. revealed the upregulation of miR-27a* (the passenger strand) in PBMCs and natural killer (NK)-cell subsets collected from patients with SLE relative to healthy subjects [26]. They found that forced expression of miR-27a* through gain/loss-of-function experiments could impact the expression of “NKG2D”, an activating receptor of NK cells, in SLE patients. Additionally, a significant negative correlation was found between miR-27a* expression in PBMCs of SLE patients and disease activity index (SLEDAI) scores, implying that this type of microRNA could be involved in SLE pathogenesis [26]. By screening B-cell-related miRNAs in the plasma of SLE patients using a customized qRT-PCR miRNA array, Zhang and colleagues demonstrated the diagnostic value of the differential expression of miR-27a with 13 other dysregulated miRNAs in discriminating SLE patients from healthy controls. Furthermore, they found that miR-27a had an area under curve = 0.873, with diagnostic sensitivity = 0.867 and specificity = 0.773 to distinguish SLE patients from patients with rheumatoid arthritis [34]. These findings further support the possibly essential role of miR-27a in the etiopathology of SLE.

**Figure 2:**
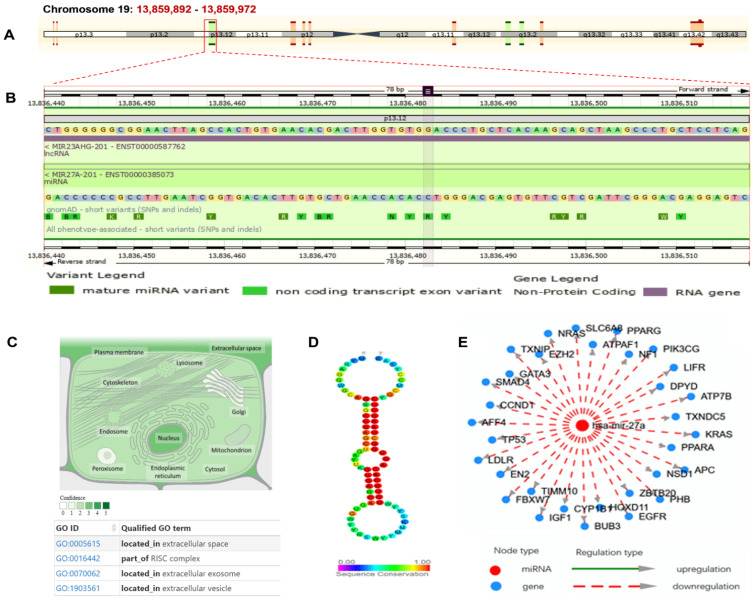
Structural analysis of microRNA27a (gene name: MIR27A) and related targets. (**A**) MIR27A is located on the short arm of chromosome 19:13,836,440–13,836,517 on the reverse strand according to the GRCh38.p14 assembly. (**B**) MIR27A is transcribed into a 78bp miRNA27a-201 transcript (ENSG00000207808). The studied noncoding transcript exon variant rs11671784G>A (ENST00000385073.1: n.36C>T) is located at position 19:13,836,482 (highlighted) on DNA gene sequence and nucleotide 36 (out of 78) of miR-27a. This variant also overlaps with the host gene MIR23AHG, which encodes long noncoding RNA (http://asia.ensembl.org/). (**C**) The subcellular distribution of miR-27a. The color degree is related to its abundance (https://www.genecards.org/). (**D**) Conserved secondary structure of pre-miR27a depicted from the noncoding RNA family database (https://rfam.org/family/RF00644#tabview). (**E**) Gene targets of miR-27a as depicted from the human microRNA disease association database (HMDD v3.0) (http://www.cuilab.cn/hmdd) (all databases were last accessed 20 December 2022). Go: gene ontology; miRNA: microRNA; RISC: RNA-induced silencing complex.

The human MiR-27a gene (MIR27A; Gene ID: 407018) is located along the short arm of chromosome 19 (Ch:19p13.12), spanning 78 base pairs (bp) (genomic coordinates at 19:13,836,440–13,836,517) on the reverse strand within the “miRNA-23∼27∼24 cluster”, according to the “Human Genome Assembly; GRCh38.p14” (https://www.ncbi.nlm.nih.gov/gene/407018) (accessed 15 December 2022) (Figure 2A). This gene is transcribed into a single 78 bp microRNA 27a (Figure 2B) and has been found predominantly intracellularly in the nucleus and extracellularly in the circulating exosomes and vesicles (Figure 2C). According to the human microRNA disease associations database (HMDD v3.0) (http://www.cuilab.cn/hmdd) (last accessed 20 December 2022), this microRNA can bind and downregulate several target genes, such as “tumor protein p53 (*TP53*)*,* Cytochrome P450 Family 1 Subfamily B Member 1 (*CYP1B1*)*,* Adenomatosis Polyposis Coli (*APC*), Engrailed Homeobox 2 (*EN2*), *GATA* Binding Protein 3 (*GATA3*), SMAD Family Member 4 (*SMAD*4), Prohibitin 1 (*PHB*)*,* Low Density Lipoprotein Receptor (*LDLR*), Nuclear Receptor Binding SET Domain Protein 1 (*NSD1*), Dihydropyrimidine Dehydrogenase (*DPYD*), Thioredoxin Interacting Protein (*TXNIP*), Translocase Of Inner Mitochondrial Membrane 10 (*TIMM10*)*,* F-Box And WD Repeat Domain Containing 7 (*FBXW7*), Insulin Like Growth Factor 1 (*IGF1*), Neuroblastoma RAS Viral Oncogene Homolog GTPase (*NRAS*), ALF Transcription Elongation Factor 4 *(AFF4*), Zinc Finger And BTB Domain Containing 20 (*ZBTB20*), Phosphatidylinositol-4,5-Bisphosphate 3-Kinase Catalytic Subunit Gamma (*PIK3CG*), Peroxisome Proliferator Activated Receptor Alpha and gamma (*PPARA/G*), *Cyclin D1* (*CCND1*), Kirsten Rat Sarcoma Viral Oncogene Homolog (*KRAS*), Leukemia Inhibitory Factor Receptor (*LIFR*), Neurofibromin 1 (*NF1*), Budding Uninhibited By Benzimidazoles 3, Yeast-Homolog Mitotic Checkpoint Protein (*BUB3*), Homeobox D11 (*HOXD11*), Epidermal Growth Factor Receptor (*EGFR*), ATPase Copper Transporting Beta (*ATP7B*), ATP Synthase Mitochondrial F1 Complex Assembly Factor 1 (*ATPAF1*), Solute Carrier Family 6 Member 8 (*SLC6A8*), Enhancer Of Zeste 2 Polycomb Repressive Complex 2 Subunit (*EZH2*), and Thioredoxin Domain Containing 5 (*TXNDC5*)” (Figure 2E). Many of these target genes have been implicated in several immune-related process and disorders [27,35,36,37,38,39,40,41,42,43,44,45,46,47,48,49,50,51,52,53,54].

Single-nucleotide polymorphisms (SNPs) within miRNA genes were previously reported to be associated with susceptibility to several diseases, including autoimmune disorders [55,56,57], and can alter the expression and/or maturation of miRNA and ultimately affect its functioning [58,59,60,61]. The MIR27A rs11671784G>A variant has been identified and studied for its potential role in various diseases and cancers [62,63,64]. For example, carriers of rs11671784 A have significantly reduced gastric cancer risk and lymphatic invasion [64,65], and the “G allele” has been reported to have a more substantial impact than the A allele in promoting bladder cancer chemosensitivity [66]. Furthermore, this SNP was significantly associated with “age-related macular degeneration” [62]. However, no studies have explored the association between this variant and SLE. Therefore, we designed the present study to test the association of the rs11671784 variant with SLE susceptibility and/or phenotype.

## 2. Materials and Methods

### 2.1. Study Participants

A total of 163 adult SLE patients and 163 unrelated age- and sex-matched controls were enrolled in this study. Patients were recruited from the Rheumatology outpatient clinics of Suez Canal University Hospitals, Ismailia. They were diagnosed and assessed according to the “European League Against Rheumatism/American College of Rheumatology” diagnostic criteria for SLE [67]. A thorough review of their clinical assessment sheets was performed to determine disease severity, therapeutic history, and comorbidities. Patients with a history of other autoimmune disorders (e.g., rheumatoid arthritis, alopecia areata, vitiligo, psoriasis, multiple sclerosis, myasthenia gravis, and inflammatory bowel disease) or chronic diseases (e.g., endocrine disorders or malignancies), or a history of long-term treatment were excluded. Laboratory data, including quantification of proteinuria in 24 h, serum creatinine and blood urea levels, type and titer of antinuclear antibodies (ANA-anti DNA), and serum complement levels (C3 and C4), were collected at the time of consent. Controls should have no history of autoimmune diseases or chronic disorders. Renal involvement was defined as an increase in proteinuria (>150 mg/24 h), an increase in serum creatinine (>1.4 mg/dL), or both [67]. The “SLE Disease Activity Index (SLEDAI) score” was used to classify patients according to disease activity into (a) score = 0, i.e., no activity; (b) score = 1:5, i.e., mild activity; (c) score = 6:10, i.e., moderate activity; (d) score = 11:19, i.e., high activity; or (E) score ≥ 20, i.e., very high activity [68].

The study was conducted in accordance with the guidelines of the Declaration of Helsinki, and written informed consent was obtained from participants before taking part.

### 2.2. MIR27A rs11671784G>A Genotyping

Five milliliters of blood was collected from each participant in an EDTA tube for hematological and molecular studies and in a plain tube for immune and biochemical studies, as detailed previously [61]. DNA was extracted from whole blood using a QIAamp DNA extraction mini kit (Cat no. 51104; Qiagen, Hilden, Germany) and assessed for concentration/purity by a “NanoDrop ND-1000 spectrophotometer” (NanoDrop Technologies, Wilmington, DE, USA). Genotyping was carried out using real-time polymerase chain reaction allelic discrimination technology on a StepOne real-time system (Applied Biosystems, Waltham, MA, USA). The applied protocol was followed blindly, regardless of the case/control status of the samples, with a final volume of 20 μL, including (a) genomic DNA (20 ng); (b) a TaqMan SNP genotyping assay mix (1 μL of the assay ID: C_176018176_10; Cat no. 4351379, Applied Biosystems, Waltham, MA, USA) to detect the transition substitution of the studied variant in the following context sequence: “GCCACTGTGAACACGACTTGGTGTG[G/A] ACCCTGCTCACAAGCAGCTAAGCCC” in which VIC/FAM-labeled probes specify the “G” and “A” alleles, respectively; (c) a TaqMan Universal PCR master mix (10 μL); and (d) nuclease-free water. Negative controls were applied in each run. The program was set at 10 min for an initial hold (95 °C), followed by a 40-cycle, two-step 15 s denaturation (95 °C) and 1 min annealing/extension (60 °C). “SDS software version 1.3.1” (Applied Biosystems, Waltham, MA, USA) was applied for allelic discrimination calling [2,69]. About 10% of the total samples were regenotyped as technical replicates, which yielded a 100% recall rate.

### 2.3. Statistical Analysis

General statistical analyses were performed with Statistical Package for Social Science (SPSS) software version 23 (IBM SPSS Statistics for Windows, Version 27.0. Armonk, NY, USA: IBM Corp). Categorical variables were compared using chi-square or Fisher’s exact tests. Student’s *t*-tests, Mann–Whitney U (MW), and Kruskal–Wallis (KW) tests were used to compare continuous variables according to data distribution/variance homogeneity, which were checked by the Shapiro–Wilk test and Levene test, respectively, to compare continuous variables. Data were expressed as mean ± standard deviation (SD). SNPstats software (version 1.24.0) was applied for genotype/allele frequency estimation as previously described [70]. Hardy–Weinberg equilibrium (HWE) testing was checked. Logistic regression analysis was applied, and adjustment for confounding parameters was considered. A two-tailed *p*-value less than 0.05 was considered statistically significant.

## 3. Results

### 3.1. Patient Characteristics

**Figure 3:**
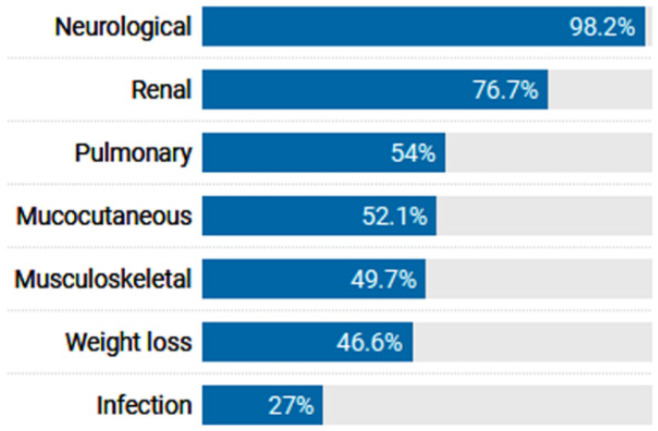
Organ involvement in SLE patients (n = 163).

This study included 163 SLE patients (147 females and 16 males) and 163 age- and sex-matched controls (148 females and 15 males). The mean age of participants was 35.6 ± 9.6 years for patients and 35.8 ± 9.9 years for controls. Fifty-eight (35.6%) cases had a positive family history of SLE. The median SLEDAI score for patients was 3.0 (IQR = 0.0–6.0). Almost all patients presented with neurological symptoms, and 76.7% of the cohort had renal involvement (Figure 3). Laboratory data of patients with SLE are summarized in Supplementary Materials Table S1.

### 3.2. Allelic Discrimination Analysis

**Figure 4:**
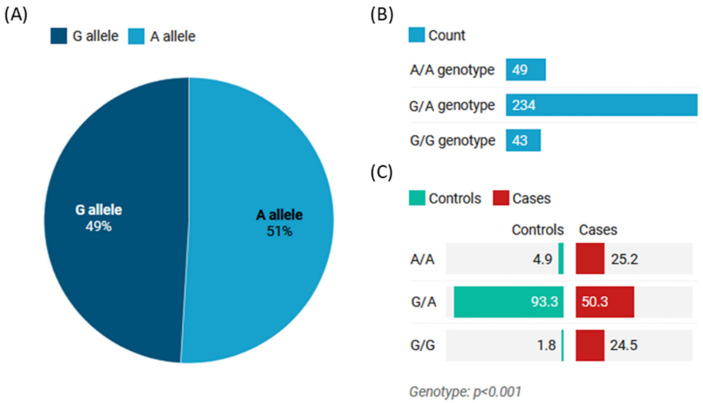
Genotype and allele frequencies of MIR27A rs11671784 (G/A) polymorphism. (**A**) The allele frequency in the study population. (**B**) The genotype frequency in the study population. (**C**) The genotype frequency in the study groups (controls vs. cases). Values are shown as a percentage. The Chi-square test was used. *p*-value < 0.05 was considered statistically significant.

In the study population (n = 326), the minor allele frequency (G allele) was 49% (n = 320). The heterozygote form was the most common genotype among the cohort, accounting for 72% of the population (n = 234), while A/A and G/G represented 15% (n = 49) and 13% (n = 43), respectively (Figure 4A). Similarly, the most prevalent genotype among cases was the G/A genotype, which was present in approximately 93.3% of cases (n = 152). In contrast, only eight and three patients had A/A and G/G genotypes, respectively. Compared with controls, the homozygote genotypes were significantly higher (A/A: 25.2% vs. 4.9% and G/G: 24.5% vs. 1.8%) in patients with SLE. In contrast, the G/A genotype of MIR27A polymorphism was less prevalent in cases (50.3% vs. 93.3%, *p* < 0.001) (Figure 4B).

The MIR27A rs11671784 variant conferred protection against the development of SLE in several genetic models, including heterozygous (G/A vs. A/A; OR = 0.10, 95% CI = 0.05–0.23), dominant (G/A + G/G vs. AA; OR = 0.15, 95% CI = 0.07–0.34), and overdominant (G/A vs. A/A + G/G; OR = 0.07, 95% CI = 0.04–0.14) models. However, the G/G genotype was associated with increased SLE risk in the recessive model (G/G vs. A/A+ G/G; OR = 17.34, 95% CI = 5.24–57.38) (Table 1).

### 3.3. MIR27A rs11671784G/A Variant Association with Clinicolaboratory Data

**Figure 5:**
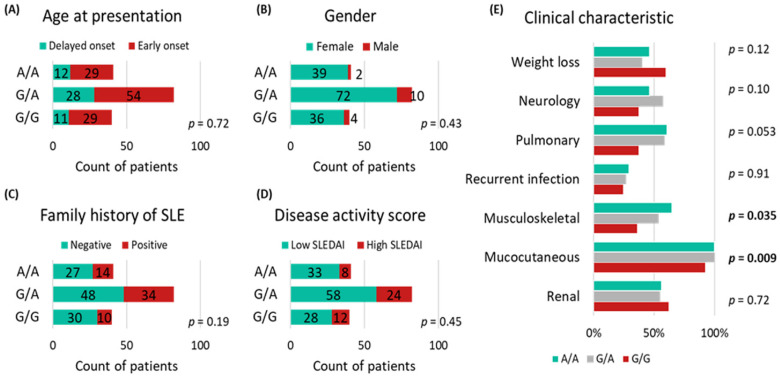
Analysis of the association of MIR27A rs11671784 (G/A) polymorphism with disease severity and clinical parameters. (**A**) Age at presentation; (**B**) gender; (**C**) family history of SLE; (**D**) disease activity score; (**E**) clinical characteristic and organ involvement. Early onset: age at diagnosis < 40 years; severe stage: SLEDAI score >6. The chi-square test was applied. Bold *p*-values < 0.05 are considered statistically significant.

**Figure 6:**
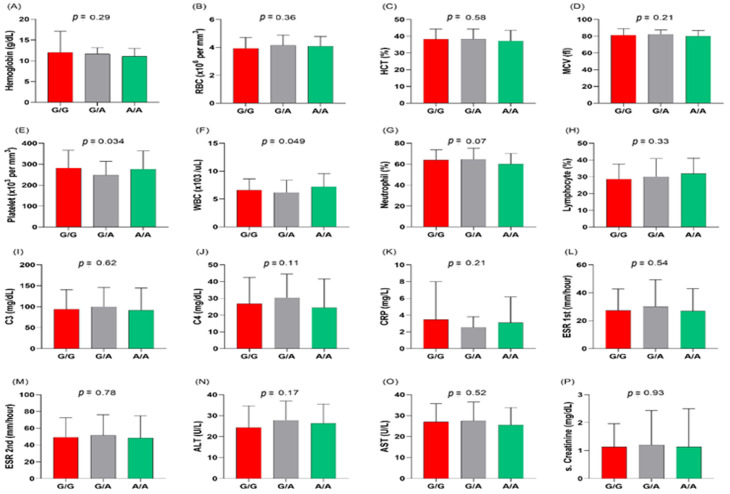
Association of the MIR27A rs11671784 (G/A) polymorphism with biochemical characteristics in patients with SLE. (**A**) Hemoglobin level (g/dL); (**B**) red blood cell count (RBC; ×10^6^ per mm^3^); (**C**) hematocrit concentration (HCT; %); (**D**) mean cell volume (MCV; fl); (**E**) platelet count (×10^3^ per mm^3^); (**F**) white blood cell count (WBC; ×10^6^ per mm^3^); (**G**) neutrophil percentage (%); (**H**) lymphocyte percentage (%); (**I**,**J**) complement 3/4 (C3/4; mg/dL); (**K**) C-reactive protein (CRP; mg/L); (**L**,**M**) erythrocyte sedimentation rate in the first/second hour (ESR 1st and 2nd; mm/hour); (**N**) alanine transaminase (ALT; U/L); (**O**) aspartate transaminase (AST; U/L); (**P**) creatinine levels (mg/dL). *p*-value < 0.05 is considered statistically significant.

Figure 5 indicates that the MIR27A rs11671784 variant is associated with musculoskeletal and mucocutaneous manifestations in patients with SLE (*p* = 0.035 and 0.009, respectively). It also shows an association with platelet and white blood cell counts (*p* = 0.034 and 0.049, respectively) (Figure 6). Otherwise, this variant does not show significant associations with other clinical and laboratory characteristics of the patients.

### 3.4. Multivariate Regression Analysis

**Figure 7:**
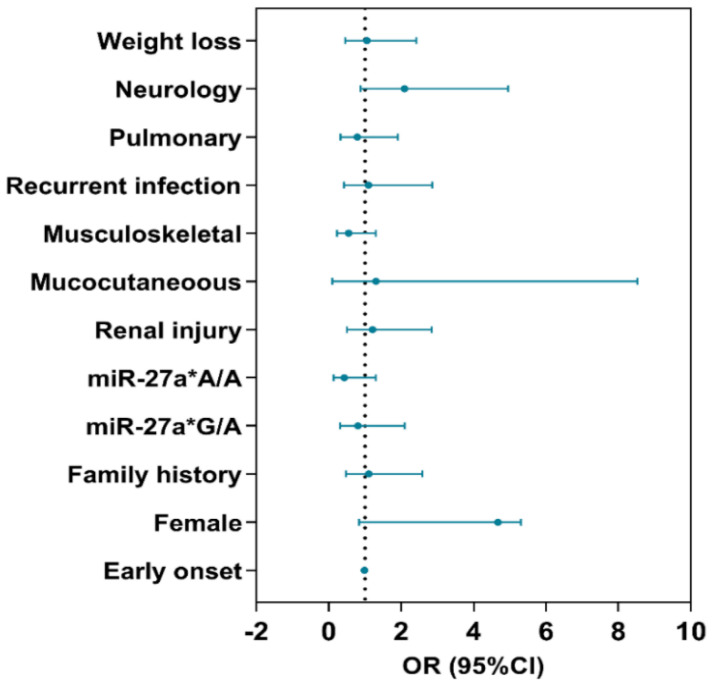
Putative risk factors for severe SLE. Multivariate regression analysis was performed, and odds ratios (ORs) and confidence interval (CIs) are reported for each variable in the forest plot.

Multivariate analysis failed to define independent predictor risk factors for the severe disease phenotype of the studied cohort with SLE, as indicated by the confidence intervals crossing the vertical line of 1 in Figure 7.

### 3.5. MIR27A Implication in SLE Etiopathology

**Figure 8:**
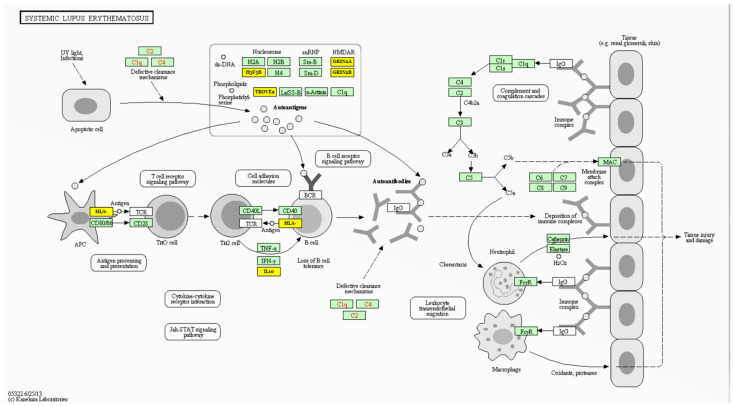
Gene targets of miR-27a in the systemic lupus erythematosus KEGG (hsa05322) pathway. The gene product targets represented here (yellow box) include the SS-A/Ro ribonucleoprotein (TROVE2), H3.3 core histone protein (H3F3B), glutamate ionotropic receptor NMDA type subunit 2A/B (GRIN2A/B), interleukin 10 (IL10), and human leukocyte antigen (HLA). Data source: Diana Lab tools (DIANA TOOLS-Reverse Mirpath (grnet.gr) (last accessed on 20 December 2022).

Figure 8 and Table S2 show the experimentally validated gene targets of miR-27a-5p in the SLE Kyoto Encyclopedia of Genes and Genomes (KEGG) pathway (hsa05322), which include several histone variants (e.g., H3F3B) that are involved in the autoantigen clearance/tolerance mechanism, the “major histocompatibility complex class II (MHCII)”, and the “HLA class II histocompatibility antigen-DO alpha chain (HLA-DOA)”, which are implicated in antigen presentation, RNA-binding proteins (e.g., TROVE2 and SNRPB), glutamate ionotropic receptor NMDA type subunit 2A/B (GRIN2A/B), cytokines (i.e., IL10), and cluster of differentiation 28 (CD28) [71,72,73].

**Figure 9:**
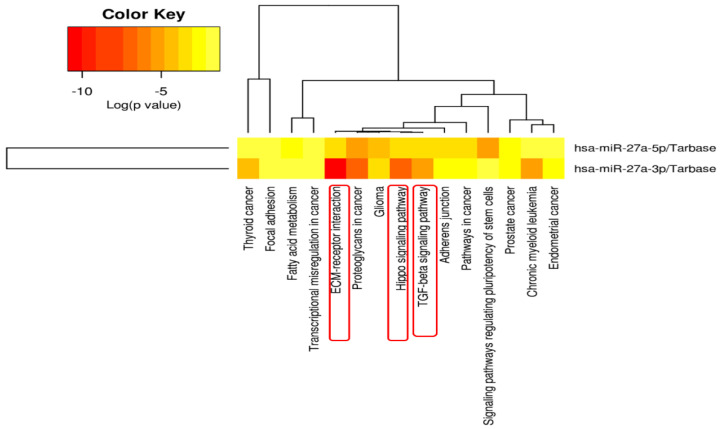
Functional enrichment analysis of MIR27A target genes. The color key bar indicates the log (*p*-value) of the enrichment analysis. The direction towards the red color indicates more significance, as the log *p*  <  0.05 is equivalent to *p*  <  −1.30. The significant pathways that may play a role in SLE etiopathology are marked with a red box. Data source: Diana lab tools (Last accessed on 20 December 2022). ECM: extracellular matrix; hsa: homo Sapiens = human; TGF: transforming growth factor.

KEGG pathway enrichment analysis for MIR27A showed significant implications of its targets in extracellular matrix–receptor interaction, Hippo signaling, and transforming growth factor-beta signaling pathways (Figure 9).

## 4. Discussion

Recent evidence suggests that miRNA variants are associated with susceptibility to several autoimmune diseases, including SLE [74]. For example, the “rs3746444” variant of miR-499 has been associated with an increased risk of SLE [75], rheumatoid arthritis [76], and other autoimmune diseases [77,78]. The miR-146a “rs57095329” variant was associated with increased SLE risk in East Asian regions [74,79,80]. The miR-149 rs2292832 polymorphism may confer susceptibility to Kawasaki disease [81], allergic rhinitis, and comorbid asthma in Chinese children [82]. The association of the miRNA-34a rs2666433 variant with SLE susceptibility and the miR-17 rs4284505 variant with susceptibility and severity of SLE were also evident in the present cohort [2,61]. These SNPs can impact biogenesis and/or dysregulate miRNAs, with a subsequent influence on immune development/differentiation or response, leading to loss of immune tolerance and autoimmunity [83,84].

The role of miR-27a in SLE has been studied extensively in recent years. Studies have shown that circulating plasma miR-27a is dysregulated in SLE patients compared to healthy controls, with an area under the curve (AUC) = 0.948, diagnostic sensitivity = 0.818, and specificity = 1.000 [34]. Furthermore, miR-27a is involved in the regulation of genes associated with SLE, such as interferon (IFN)-γ [85], interleukin (IL)-10 [86], and transforming growth factor (TGF)-β [87,88], among others. MiR-27a regulates these genes by binding to their 3′UTR and inhibiting their expression. The role of miR-27a in SLE has been further investigated in the peripheral blood mononuclear cells and natural killer cells of patients with SLE compared to controls [26]. In this latter study, the authors identified aberrant expression of miR27a in the isolated cells and found that forcing miR27a expression enhances NKG2D (natural killer activating cell receptor) mRNA expression and could have a role in SLE etiopathology.

Our in silico analysis confirmed the implication of miR-27a in the SLE pathway by targeting several genes coding for variable histone family proteins, RNA-binding proteins, and several immune-response-related proteins, such as HLA class II histocompatibility antigen and CD28, which can modulate antigen processing and presentation by immune cells, autoantigen production, and the clearance mechanism, as depicted in Figure 8. Furthermore, enrichment analysis of miR-27a target genes shows significant involvement of miR-27a in potential pathways that may play a role in SLE etiopathology, such as extracellular matrix–receptor interaction [89], Hippo signaling [90], and transforming growth factor-beta signaling pathways [91].

In addition to its role in SLE, miR-27a has also been found to be involved in other autoimmune diseases, such as rheumatoid arthritis [92] and systemic sclerosis [93]. In a placebo-controlled trial, it was shown that miR-27a is a potential biomarker for the favorable response to methotrexate/disease-modifying antirheumatic drug combination therapy in patients with rheumatoid arthritis [94].

In the present study, we found that the homozygous A/A and G/G genotypes of the MIR27A variant are more common in individuals with SLE than in healthy individuals and that the G/G genotype was associated with an increased risk of developing SLE in the recessive model. In contrast, the G/A genotype revealed a protective effect against the development of SLE. The exact mechanism by which the MIR27A polymorphism could be associated with the risk of developing SLE is not yet known, but it can be speculated to affect the expression level of mature microRNA, which can impact the target genes involved in the immune system. By running the HaploReg v3 tool (https://pubs.broadinstitute.org/mammals/haploreg/haploreg_v3.phpto) (last accessed on 20 December 2022) [95] to predict the effect of the studied variant, we found that this SNP can disrupt the Brachyury (a T-box transcription factor T) and Eomes (a T-box transcription factor), as well as HNF4 (Hepatocyte Nuclear Factor 4), DNA motifs. Brachyury is involved in transcription repression by RNA polymerase II (https://www.ncbi.nlm.nih.gov/gene/20997) is a generated (last accessed 20 December 2022), and Eomes is implicated in CD8 T-cell/natural killer cell differentiation [96] and plays a substantial role in regulating cytotoxic function/development and survival of immune cells [97].

Previous evidence also explained the role of the MIR27A rs11671784 variant in other diseases by influencing the miR-27a maturation and/or expression levels. For example, Katayama et al. reported that this variant can downregulate mature miR-27a with subsequent increased expression of its target genes in bladder cancer cells [66]. Others suggested that it can impact the processing efficiency of miR-27a [98]. Interestingly, Strafella and colleagues computed the minimum free energy (MFE) of a miR-27a hairpin structure, including the variant A allele, which generated a secondary structure with an “MFE = −38.76 Kcal/mol”, whereas the structure with the G allele showed an “MFE = −38.24 Kcal/mol” [62]. They concluded that the rs11671784 polymorphism located in the terminal loop of the pre-miR27a might influence the expression levels of mature miR-27a without substantially, impairing its processing and binding affinity with target mRNAs [62]. All these findings support the significant association of the studied variant with SLE susceptibility/development reported in the present study. Further mechanistic research is needed to understand the precise implications of this polymorphism for SLE.

Although the studied variant did not show significant associations with most clinicolaboratory characteristics of the patients, it was associated with musculoskeletal and mucocutaneous manifestations and showed borderline associations with platelet and white blood cell counts. Interestingly, miRNA 27a has been found to play a vital role in osteogenesis, and its expression is downregulated upon osteogenic differentiation [99]. The latter investigators revealed that grancalcin, “a regulator of osteogenesis” in human mesenchymal stem cells, is a target of miR-27a. Furthermore, the reported impact of miR-27a on the overall regulation of “matrix metalloprotease-13” and “insulin-like growth factor binding protein”, two genes involved in osteoarthritis pathophysiology and some skin disorders [25,27,28,31,100,101], could partially support the association of this variant with the identified clinical manifestations in the present SLE cohort. Additionally, miR-27a has been found to attenuate the expression of a critical regulator of hematopoiesis, the “RUNX1 transcription factor”, in K562 cells, which could impact megakaryopoiesis and differentiation [102]. This could partly explain the association of the studied variant with the hematological findings reported in the present study.

It is worth noting that besides the studied MIR27A rs11671784 variant, several genetic/epigenetic and environmental factors also participate in SLE susceptibility. Furthermore, the relatively small sample size, the cross-sectional analysis of hospital-based selected cohorts, and the lack of experimental studies to elucidate how this variant might impact the disease could all limit this work. In this sense, large-scale longitudinal studies on multiethnic populations supported with functional analyses are recommended.

## 5. Conclusions

We are the first to provide evidence that the MIR27a rs11671784 genetic variant could be associated with SLE susceptibility/risk in the studied population. The homozygous A/A and G/G genotypes of the miR-27a variant were more common in patients with SLE than in healthy individuals, and the G/G genotype was associated with increased SLE risk in the recessive model. Nevertheless, the studied variant did not show significant associations with most clinical and laboratory characteristics of the patients, although there was a significant association with musculoskeletal and mucocutaneous manifestations. The potential impact of this variant on gene stability and processing with subsequent influence on target genes related to SLE etiopathology requires future mechanistic validation studies.

## Figures and Tables

**Table 1:** Risk of systemic lupus erythematosus by genetic association models of miR-27 rs11671784 (G/A) genotypes.

Model	Genotype	Controls	Cases	Adjusted OR (95% CI)	*p*-Value
Codominant	A/A	8 (4.9%)	41 (25.1%)	1.00	<0.0001
	G/A	152 (93.2%)	82 (50.3%)	**0.10 (0.05–0.23)**	
	G/G	3 (1.8%)	40 (24.5%)	2.56 (0.63–10.36)	
Dominant	A/A	8 (4.9%)	41 (25.1%)	1.00	<0.0001
	G/A-G/G	155 (95.1%)	122 (74.8%)	**0.15 (0.07–0.34)**	
Recessive	A/A-G/A	160 (98.2%)	123 (75.5%)	1.00	<0.0001
	G/G	3 (1.8%)	40 (24.5%)	**17.34 (5.24–57.38)**	
Overdominant	A/A-G/G	11 (6.8%)	81 (49.7%)	1.00	<0.0001
	G/A	152 (93.2%)	82 (50.3%)	**0.07 (0.04–0.14)**	
Log-additive	---	---	---	1.09 (0.72–1.64)	0.68

Values are shown as numbers (%). The chi-square test was used. OR (95% CI), odds ratio, and confidence interval. *p*-value < 0.05 is considered statistically significant. Adjusted covariates: age and sex. The protective association (OR < 1) is indicated by green color, while risky association is indicated in red.

## Data Availability

All data generated in this study are included in the article.

## Supplementary Materials

The following are available online at https://www.mdpi.com/article/10.3390/life13030701/s1, Table S1: Biochemical characteristics of patients with systemic lupus erythematosus, Table S2: TargetScan predicted interactions for hsa-miR-27a-3p in the systemic lupus erythematosus (hsa05322) pathway.
