# Supplementary material for: Analysis of MIR27A (rs11671784) Variant Association with Systemic Lupus Erythematous
Source: Life (Basel). 2023 Mar 5;13(3):701. doi: 10.3390/life13030701 (PMC10058767; doi:10.3390/life13030701)
Supplement: Supplementary file 1 [file life-13-00701-s001.zip › life-2204018-supplementary.pdf]

Supplementary Table S1. Laboratory-related parameters of patients with systemic lupus erythematosus.

**Laboratory test**	**Value**
Hemoglobin (g/dL)	11.6 ± 2.9
RBC (x10^6 per mm^3)	4.1 ± 0.7
HCT (%)	38.0 ± 6.1
MCV (fl)	81.5 ± 6.3
Platelet count (x10^3 per mm^3)	263.9 ± 77.4
WBC (x10^3 /uL)	6.5 ± 2.2
Neutrophil (%)	63.4 ± 10.4
Lymphocyte (%)	30.2 ± 10.0
C3 (mg/dL)	96.3 ± 47.9
C4 (mg/dL)	28.0 ± 15.5
CRP (mg/L)	2.4 (1.5-3.2)
ESR first hour (mm/hour)	26.8 ± 13.6
ALT (U/L)	26.6 ± 9.6
AST (U/L)	26.9 ± 8.8
Serum creatinine (mg/dL)	1.0 (0.8-1.2)
Urea (mg/dL)	35.0 ± 11.9
Serum albumin (g/dL)	4.3 ± 0.6

Data are presented as mean ± SD. RBC: red blood cell, HCT: hematocrit, MCV: mean cell volume, WBC: white blood cell, C3/4: complement 3/4, CRP: C-reactive protein, ESR: erythrocyte sedimentation rate, ALT, alanine transaminase; AST, aspartate transaminase.

Supplementary Table S2. TargetScan Predicted Interactions for hsa-miR-27a-3p in Systemic lupus erythematosus (hsa05322) pathway

No.	Gene Name	Gene Ensembl id	Context Score	Conservation Score
**1.**	**TROVE2**	ENSG00000116747	-0.188	0.795
**2.**	**HLA-DOA**	ENSG00000204252	-0.107	0.735
**3.**	**CD28**	ENSG00000178562	-0.123	0.371
**4.**	**H3F3B**	ENSG00000132475	-0.119	0.791
**5.**	**GRIN2A**	ENSG00000183454	-0.168	0.414
**6.**	**IL10**	ENSG00000136634	-0.100	0.607
**7.**	**GRIN2B**	ENSG00000273079	0.164	0.291

Data retrieved from https://dianalab.e-ce.uth.gr/ (last accessed 20 Dec 2022)
